# Germline Mutational Landscape in Chinese Patients With Advanced Breast Cancer

**DOI:** 10.3389/fonc.2022.745796

**Published:** 2022-04-13

**Authors:** Jiayang Zhang, Nan Wang, Tiantian Zheng, Tan Lu, Ruyan Zhang, Ran Ran, Kun Li, Yong Huang, Feng Xie, Yue Zhang, Shidong Jia, Jianjun Yu, Huiping Li

**Affiliations:** ^1^ Key Laboratory of Carcinogenesis and Translational Research (Ministry of Education/Beijing), Department of Breast Oncology, Peking University Cancer Hospital and Institute, Beijing, China; ^2^ Huidu Shanghai Medical Sciences, Shanghai, China

**Keywords:** breast cancer, germline mutations, DNA-damage repair pathway, next-generation sequencing, prognosis

## Abstract

**Background:**

Genetic testing for breast cancer (BC) patients may shift the paradigm towards more personalized management and treatment strategies. While gene alterations may be ethnic-specific in breast cancer, our understanding of genetic epidemiology of BC remains mainly driven by data from Caucasian populations and further limited to selected handful of genes.

**Methods:**

We collected whole blood samples from 356 BC patients at metastatic first line BC and primary stage IV disease at Beijing Cancer Hospital between Jan. 2013 to Dec. 2019. A comprehensive 600-gene cancer panel was used to detect germline variants in the covered genes with a median 300x sequencing depth. Variants were classified into pathogenic, likely pathogenic, variant of uncertain significance, likely benign and benign groups according to the ACMG/AMP Standards and Guidelines. Pathogenic and likely pathogenic variants were considered as deleterious mutations.

**Results:**

The median age of 356 BC patients was 49 years (range, 21-87 years) at the first diagnosis of BC. Deleterious germline mutations across 48 cancer-related genes were identified in 21.6% (77/356) of the patients. The most prevalent mutations were BRCA1/2 mutations (7.0%), followed by ATM and RAD50 mutations (1.4% each). In addition, patients with family history were more likely to carry BRCA1 mutations (P=0.04). Moreover, patients with triple-negative breast cancer (TNBC) were more likely to harbor BRCA1 mutations than those with HR+ or HER2+ breast cancer (P=0.006). While there was no significant survival difference observed in BRCA1/2 carriers relative to non-carriers, patients with DNA damage repair (DDR) gene mutations (mostly frequently BRCA, ATM, RAD50) had worse disease-free survival (P=0.02).

**Conclusions:**

The most prevalent germline mutations in a large cohort of Chinese patients with advanced BC were BRCA1/2 mutations, followed by ATM and RAD50 mutations. In total, approximately 16.0% (57/356) of patients carry deleterious mutations in DDR pathway. Patients with breast or ovarian cancer family history were more likely to carry BRCA1/2 mutations, and ones with DDR mutations had worse survival. These findings suggest that DDR mutations are prevalent in Chinese BC patients who may potentially benefit from treatment with Poly (ADP-ribose) polymerase inhibitors.

## Introduction

The incidence of breast cancer (BC) has been rising and approaching 36.1/100,000 in China (1). Actionable genetic mutations account for 5-10% of BC occurrence (2–4). Genetic testing for BC patients might change traditional management paradigms to encompass personalized treatment strategies (5, 6). For instance, genetic testing for germline BRCA1/2 mutations has evolved to be a part of the standard clinical practice in patients with triple negative breast cancer (TNBC) (5, 7, 8). As a consequence, patients harboring deleterious mutations of BRCA1/BRCA2 genes are identified who may be highly sensitive to treatment with DNA-damaging agents such as platinum-based chemotherapy agents, topoisomerase II inhibitors and recent poly (ADP‐ribose) polymerase (PARP) inhibitors (7, 9). Multiple genes are associated with hereditary BC, most of which are involved in DNA damage repair pathways such as homologous recombination repair (HRR) and mismatch repair (MMR) (4, 9–11). Mutation carriers of BRCA1, BRCA2, CDH1, CHEK2, MSH2, and ATM account for 10% of all BC patients (2).

It is becoming increasingly evident that germline BC mutations may vary across ethnicities (12, 13). For example, founder mutations in BRCA1 (187delAG and 5385insC) and BRCA2 (6174delT) constitute more than 90% of mutations in Ashkenazi Jews, but occur less frequently in other populations (14–16). Distinct BRCA1 c.5470_5477delTGCCCAAT and BRCA1 c.981_982delAT occur frequently in Chinese individuals, suggesting that they are potential founder mutations in the Chinese population (5, 10, 13). The frequencies of gene mutations also differ by ethnic groups. The frequency of BRCA1/2 gene mutations in Ashkenazi Jews is approximately 11.1% (16), but is lower in other populations, with an estimate of 5.3% in unselected Chinese patients (4) and 6.0% in unselected European patients (8), respectively. The frequencies in other breast cancer susceptibility genes such as CHEK2 and ATM are lower in East Asian populations than that of European populations (8, 10).

Currently the genetic epidemiology of BC is mainly based on data from Caucasian populations (17). Being the largest non-European population, the Chinese population is further diversified across regions (18). Whether currently available genetic information can guide clinical practice in the Chinese population remains underexplored. To elucidate the landscape of germline mutations in Chinese BC patients, we retrospectively analyzed the clinical and genetic data from patients treated at the Department of Breast Oncology, Peking University Cancer Hospital from Jan. 2013 to Dec. 2019. Gene mutations, age of onset, family history, phenotype and clinical outcomes were analyzed.

## Patients and Methods

### Patients

We selected patients with advanced breast cancer treated at the Department of Breast Oncology, Peking University Cancer Hospital from Jan. 2013 to Dec. 2019, who according to the following inclusion criteria: 1) Received a diagnosis of pathologically-confirmed advanced breast cancer, 2) Did not receive any prior chemotherapy for metastatic disease; 3) Had at least one measurable lesion detected by imaging according to the Response Evaluation Criteria in Solid Tumors (RECIST) version 1.1; and 4) Had a performance status score of Eastern Cooperative Oncology Group (ECOG) =0, 1).

The study was approved by the ethics committee of Peking University Cancer Hospital (No. 2017KT40). Follow-up was routinely performed by regular inpatient, outpatient or telephone visit every 8-12 weeks. The last follow-up was March 31, 2020. Disease-free survival (DFS) was defined as the time from surgery to first recurrence or metastasis, or death from any cause. Overall survival (OS) was calculated as the date of diagnosis of breast cancer until the last follow up or date of death.

### Next-Generation Sequencing (NGS)

#### DNA Extraction

Genomic DNA (gDNA) was extracted from peripheral blood mononuclear cells isolated from whole-blood samples. 250ng of gDNA was enzymatically fragmented to generate a main peak at ~250 bp and were further purified using AMPure XP beads as per the manufacturer’s instructions.

Forty nanograms of fragmented gDNA were subjected to library construction, including end-repair dA-tailing and adapter ligation. Ligated library fragments with appropriate adapters were amplified *via* PCR. The amplified DNA libraries were then checked using Bioanalyzer 2100, and samples with yields > 700 ng (up to 2ug) were proceeded to hybrid capture.

#### Library Preparation, Enrichment and NGS Sequencing

Library capture was conducted using Biotin-labeled DNA probes (Thermo Fisher Scientific, Weltham, MA, USA). The library was hybridized using a large 600-gene PredicineATLAS cancer panel (please see **Appendix A** for detailed gene list) overnight and captured on Dynabeads M-270 Streptavidin (Thermo Fisher Scientific, Weltham, MA, USA). The unbound fragments were washed away, and the enriched fragments were amplified *via* PCR amplification. For library preparation, the purified product was checked using a Bioanalyzer 2100 and loaded into a NovoSeq 6000 (Illumina, San Diego, CA, USA) for NGS using paired-end 2×150 bp sequencing kits.

#### NGS Sequencing Data Analysis

Data were analyzed using the Huidu in-house analysis pipeline, which starts from the raw sequencing database call files (BCL) and outputs the final mutation calls. Briefly, the pipeline first performed an adapter trim, barcode checking, and correction. Cleaned paired FASTQ files were aligned to human reference genome build hg19 using Burrows-Wheeler Aligner. Consensus bam files were then derived by merging paired-end reads originated from the same molecules (based on mapping location and unique molecular identifiers) as single strand fragments. Single strand fragments from the same double strand DNA molecules were further merged as double stranded for suppressing sequencing and PCR errors. NGS quality-checking was performed by examining the percentage of targeted regions with >50x unique consensus coverage. Samples with <95% regions having >50x unique coverage were deemed to be QC-failed.

Candidate variants, consisting of point mutations, small insertions and deletions, were identified using the inhouse developed pipeline across the targeted regions covered in the PredicineATLAS panel. Candidate variants with low base quality, mapping scores, and other quality metrics were filtered. Candidate variants in repeat regions were also excluded. Next, variants are classified into pathogenic, likely pathogenic, variant of uncertain significance, likely benign and benign according to the American College of Medical Genetics and Genomics and the Association for Molecular Pathology (ACMG/AMP) standards and guidelines. Pathogenic and likely pathogenic variants are considered as deleterious mutations. Variants annotated as benign or likely benign were filtered. Variants present in public databases of common germline variants, including 1000 genomes, ExAC, gnomAD, and KAVIAR, with population allele frequency >5% were also filtered unless they were annotated as pathologic or likely pathologic. Variants with allele frequency <15% were then further filtered out.

### Statistical Analysis

Statistical analyses were carried out using R (http://www.r-project.org). Categorical data are presented as numbers and percentages and continuous data as medians and ranges. Fisher’s exact test was used for comparison of categorical variables. DFS, OS in association with gene alterations were estimated using the Kaplan–Meier method and statistical significance was calculated based on the log-rank test. A multivariate CoxPH regression model was also performed to adjust effects of clinical co-variables. All *p* values were two sided and P < 0.05 was considered to denote statistical significance.

## Results

### Survival Outcome in Association With Patient Clinical Characteristics

**Figure 1:**
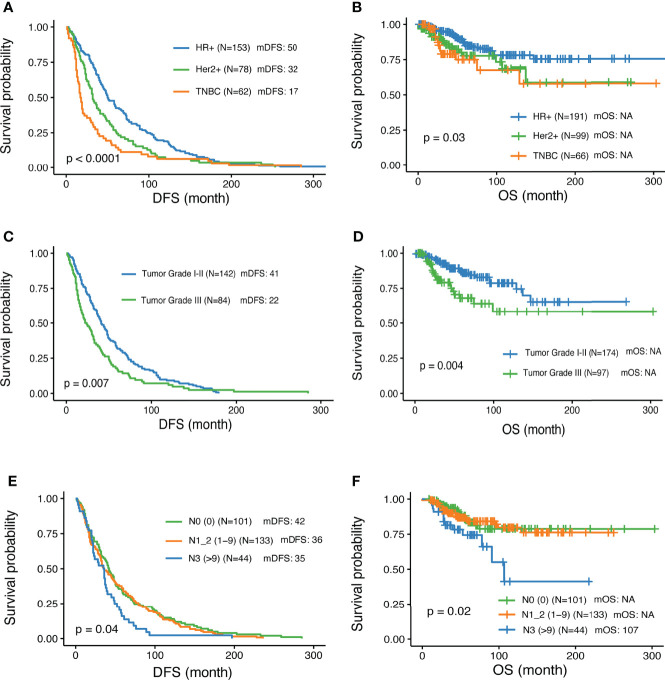
Associations of clinical parameters with disease-free survival and overall survival. **(A, B)** Significantly different outcome shown among breast cancer IHC subtypes for disease-free survival (DFS) and overall survival (OS) respectively. **(C, D)** Significantly different outcome associated with tumor grade for DFS and OS respectively. **(E, F)** Significantly different outcome associated with lymph node groups for DFS and OS respectively.

A total of 356 patients met the inclusion criteria and were enrolled in the study. Among them, 191 were diagnosed with HR+ HER2- disease by IHC; 99 with HER2+, and 66 with triple-negative breast cancer (TNBC). The clinical characteristics were summarized in **Table 1**. The median follow-up time was 52 months (range 4 – 356 months). The median age at the time of diagnosis of all the patients was 49, ranging from 21 to 87 years old. All patients relapsed and 60 patients died during the follow-up period. Disease-free survival (DFS) and overall survival (OS) were analyzed in this study. Overall, among the different IHC subtypes, HR+ patients showed the most favorable DFS, followed by Her2+, and TNBC subtypes. The HR+ subgroup of patients had a median DFS (mDFS, 50m) that was longer than HER2+ (32m) and TNBC (17m) (P<0.0001) (**Figure 1A**), whereas median OS was not reached for any of the subtypes yet (P<0.03) (**Figure 1B**). Higher tumor grade compared with lower grade was associated with worse mDFS (22 vs 41m; P=0.008, HR=1.45, 95% CI: 1.1-1.91) and OS (P=0.006, HR=2.24, 95% CI: 1.27-3.96) (**Table 1** and **Figures 1C, D**). In addition, a higher number of axillary lymph node metastases was also associated with worse DFS (P = 0.01) (**Figure 1E**) and worse OS. Patients aged 30-40 had prolonged DFS compared to younger patients (48 vs 39 month; p=0.008, HR=0.45, 95% CI: 0.25-0.81) while older patients >50 years tended to have the worst DFS survival (**Table 1**)

**Table 1:** Analysis of clinical parameters and survival outcome.

Variables	No. of patients	Disease-Free Survival				Overall Survival from Diagnosis				
		Univariate analysis		Multivariate analysis		Univariate analysis		Multivariate analysis		
		mDFS (95%CI) (m)	p value	HR (95% CI)	p value	mOS (95%CI) (m)	p value	HR (95% CI)	p value
**Median age (range)**	49 (21-87)								
**Age at diagnosis (years)**									
≤30	16 (4.5%)	39.0 (23.9-54.1)	0.001	1		NA**	0.048	1	
30-40	68 (19.1%)	48.0 (28.3-67.7)		0.45 (0.25-0.81)	0.008	NA		0.77 (0.17-3.61)	0.74
40-50	108 (30.3%)	43.0 (32.9-53.1)		0.7 (0.4-1.2)	0.19	NA		0.86 (0.19-3.8)	0.84
>50	164 (46.1%)	35.0 (30.5-39.5)		0.89 (0.52-1.52)	0.66	147.0 (86.6-207.4)		1.73 (0.42-7.19)	0.45
**Family history**									
No	257 (72.2%)	40.0 (34.0-46.0)	0.436	1		NA	0.283	1	
Breast/ovarian cancer	35 (9.8%)	41.0 (12.8-69.2)		0.98 (0.64-1.48)	0.91	NA		1.66 (0.72-3.81)	0.23
Other cancers	64 (18.0%)	36.0 (26.4-45.6)		1.16 (0.82-1.65)	0.4	NA		1.22 (0.55-2.69)	0.62
**Tumor grade**									
I-II	174 (48.9%)	41.0 (33.4-48.6)	0.000	1		NA	0.004	1	
III	97 (27.2%)	22.0 (13.0-31.0)		1.45 (1.1-1.91)	0.008	NA		2.24 (1.27-3.96)	0.006
Unknown	85 (23.9%)	67.0 (33.8-100.2)				NA			
**Lymph node**									
N0 (0)	101 (28.4%)	42.0 (33.9-50.1)	0.000	1		NA	0.000	1	
N1-2 (1-9)	133 (37.4%)	36.0 (26.8-45.2)		1.02 (0.76-1.36)	0.91	NA		1.1 (0.54-2.22)	0.80
N3 (>9)	44 (12.4%)	35.0 (22.0-48.0)		1.67 (1.11-2.52)	0.01	107 (71.8-142.2)		2.16 (0.94-4.94)	0.07
Stage IV	63 (17.7%)	0				52.0 (35.6-68.4)			
Unknown	15 (4.2%)	98.0 (70.2-125.8)				NA			
**Molecular subtype**									
TNBC	66 (18.5%)	17.0 (14.2-19.8)	0.000	1		NA	0.030	1	
HR+*	191 (53.7%)	50.0 (41.2-58.8)		0.65 (0.46-0.92)	0.01	NA		0.42 (0.2-0.89)	0.02
Her2+	99 (27.8%)	32.0 (25.3-38.7)		0.95 (0.64-1.41)	0.81	NA		0.75 (0.34-1.64)	0.46

*HR+=HR+Her2.

**NA=not applicable.

### Deleterious Mutational Landscape of Chinese Patients With Breast Cancer

**Figure 2:**
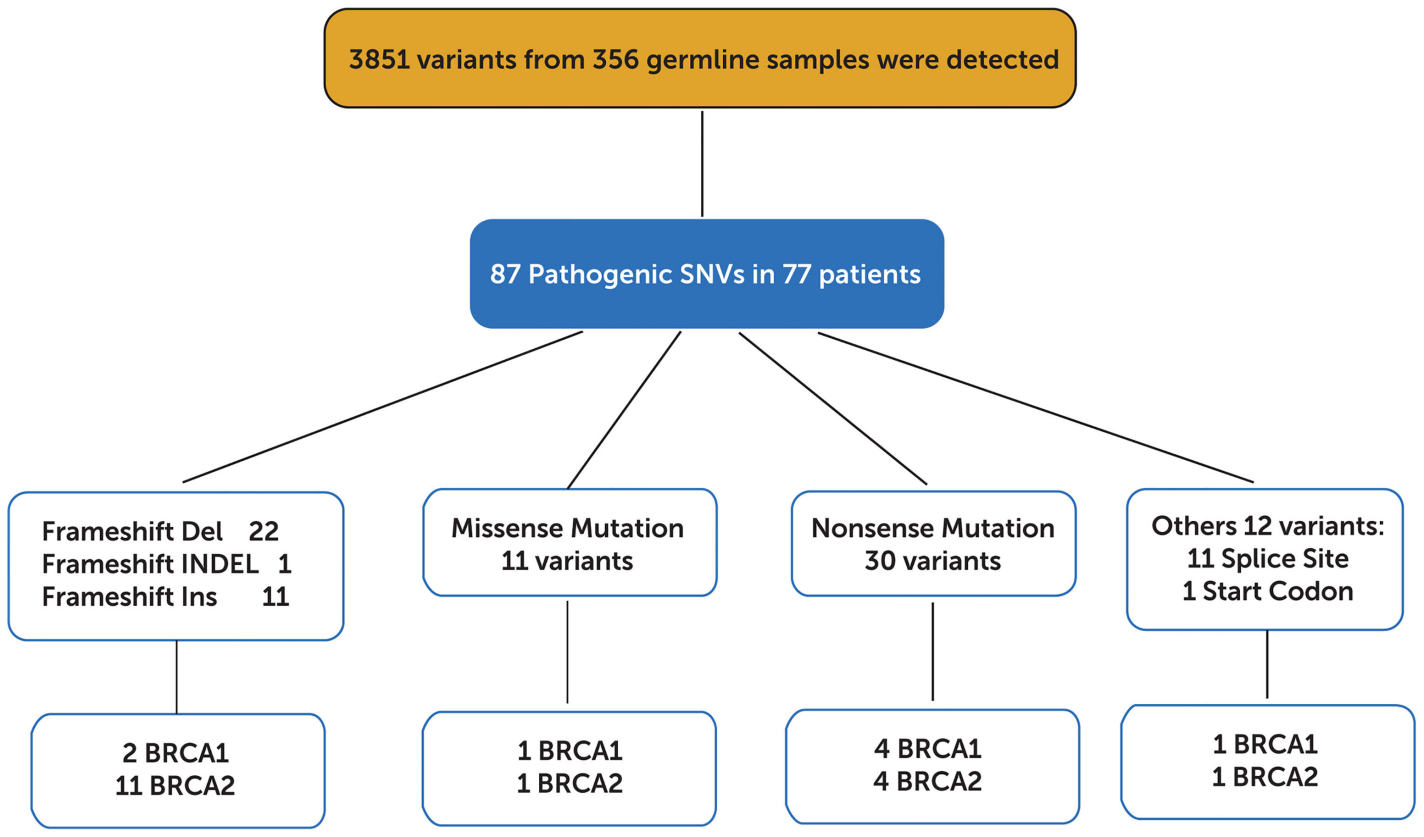
Distribution of 3851 variants detected in 356 germline samples.

**Figure 3:**
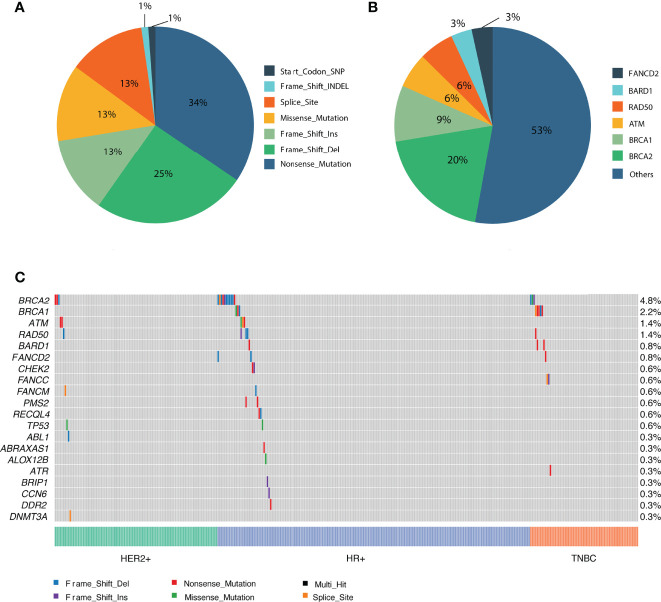
Overview of the identified pathogenic variants by PredicineATLAS panel. **(A)** Pie chart representing the variant classification of the pathogenic mutations. **(B)** Pie chart representing the overall distribution of the 87 detected pathogenic genes. **(C)** Heatmap representation of the top 20 detected pathogenic mutations across cancer subtype patients.

In total, 3585 variants were identified in 356 patients. Among them, 87 variants that are classified as (likely) pathogenic mutations according to the American College of Medical Genetics and Genomics and the Association for Molecular Pathology (ACMG/AMP) standards and guidelines (deemed as deleterious mutations) were detected in 77 patients and were thus included in the downstream statistical analyses. As shown in **Figures 2**, **3A**, the majority of them are nonsense (30) or frame-shift mutations (34), leading to early truncation of the corresponding proteins. Of these deleterious mutations, BRCA2 accounted for the highest proportion (20%), followed by BRCA1 (9%), ATM (6%), RAD50 (6%) and BARD1 (3%) (**Figure 3B**). The overall mutational landscape of the entire patient cohort revealed that 4.8% (17/356) patients carried BRCA2 mutations, and 2.2% (8/356) of the patients harbored BRCA1 mutations. Less than 2% of the patients carried deleterious mutations in other genes including ATM, RAD50, and BARD1 (**Figure 3C**).

### Association of Deleterious Mutations and Clinical Parameters

**Figure 4:**
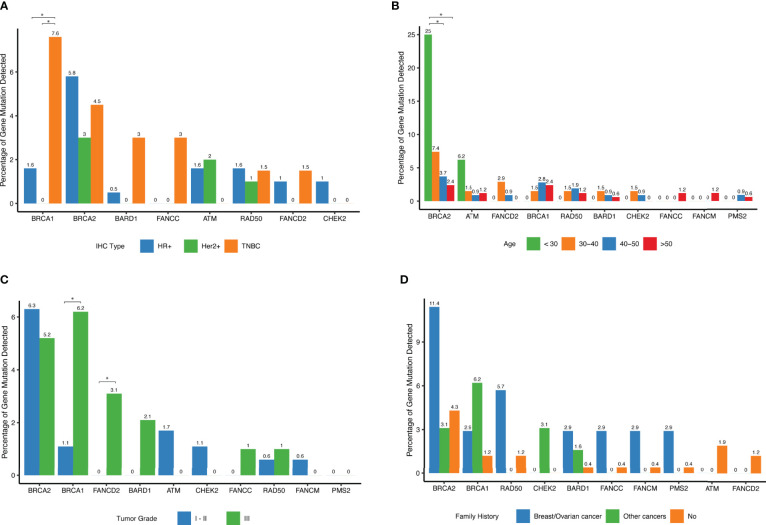
Distribution of deleterious mutations among different clinical subgroups for the selected genes. **(A)** Percent of deleterious variants detected in different IHC subtypes for a given gene. The top 10 most frequent mutated genes are shown. **(B)** Percent of deleterious variants detected in different age groups for a given gene. The top 10 most frequent mutated genes are shown. **(C)** Percent of deleterious variants detected in different tumor grade groups for each of the top 10 mutated genes. **(D)** Percent of deleterious variants detected in different family history groups for a given gene. The top 10 most frequent mutated genes are shown.

We explored associations between detected mutations and patients’ clinical parameters. While BRCA2 mutations were the most frequently detected germline mutations in this cohort, there was no significant difference among different IHC subtypes. In contrast, BRCA1 mutations tended to have higher occurrence rate among patients with triple-negative breast cancer (**Figure 4A**). No significant difference between subtypes was observed for the other genes. Notably, among young patients diagnosed with breast cancer below the age of 30, approximately 25% of them carried BRCA2 deleterious mutations and 6.2% of them harbored ATM mutations, significantly higher than in women diagnosed ≥ 40 years (P=0.002), (**Table 2** and **Figure 4B**). Interestingly, there was no significant difference among different age groups for mutational prevalence of BRCA1 and other genes (**Table 2**).

**Table 2:** Comparison of genomic alterations between BRCA1/2 mutation carriers and noncarrier among different prognostic clinical variables.

Variables	BRCA1		P value	BRCA2		P value
	Noncarriers	Carriers		Noncarriers	Carriers	
	N=348 (n(%))	N=8 (n(%))		N=339 (n(%))	N=17 (n(%))	
**Age at diagnosis (year)**						
Mean (SD)	48.8 (11.2)	48.8 (8.31)	0.99	49.2 (11.0)	41.1 (12.0)	0.003
Median (range)	49.0 [21.0, 87.0]	50.0 [35.0, 62.0]		50.0 [26.0, 87.0]	40.0 [21.0, 62.0]	
≤30	16 (4.6%)	0 (0%)	1	12 (3.5%)	4 (23.5%)	0.003
30-40	67 (19.3%)	1 (12.5%)		63 (18.6%)	5 (29.4%)	
40-50	105 (30.2%)	3 (37.5%)		104 (30.7%)	4 (23.5%)	
>50	160 (46.0%)	4 (50.0%)		160 (47.2%)	4 (23.5%)	
**Family history**						
Breast/ovarian cancer	34 (9.8%)	1 (12.5%)	0.04	31 (9.1%)	4 (23.5%)	0.17
Other cancers	60 (17.2%)	4 (50.0%)		62 (18.3%)	2 (11.8%)	
No	254 (73.0%)	3 (37.5%)		246 (72.6%)	11 (64.7%)	
**Tumor size**						
T0-1 (≤2cm)	111 (31.9%)	2 (25.0%)	1	107 (31.6%)	6 (35.3%)	0.62
T2-4 (>2cm)	151 (43.4%)	3 (37.5%)		143 (42.2%)	11 (64.7%)	
Stage IV	60 (17.2%)	3 (37.5%)		63 (18.6%)	0 (0%)	
Unknown	26 (7.5%)	0 (0%)		26 (7.7%)	0 (0%)	
**Histology of tumor**						
IDC	297 (85.3%)	6 (75.0%)	0.44	287 (84.7%)	16 (94.1%)	0.84
ILC	13 (3.7%)	0 (0%)		13 (3.8%)	0 (0%)	
Others	38 (10.9%)	2 (25.0%)		39 (11.5%)	1 (5.9%)	
**Tumor grade**						
I-II	172 (49.4%)	2 (25.0%)	0.03	163 (48.1%)	11 (64.7%)	0.79
III	91 (26.1%)	6 (75.0%)		92 (27.1%)	5 (29.4%)	
Unknown	85 (24.4%)	0 (0%)		84 (24.8%)	1 (5.9%)	
**Ki67 index**						
≤14%	36 (10.3%)	0 (0%)	1	33 (9.7%)	3 (17.6%)	0.44
>14%	261 (75.0%)	7 (87.5%)		254 (74.9%)	14 (82.4%)	
Unknown	51 (14.7%)	1 (12.5%)		52 (15.3%)	0 (0%)	
**Lymph node(count)**						
N0 (0)	100 (28.7%)	1 (12.5%)	0.47	92 (27.1%)	9 (52.9%)	0.002
N1-2 (1-9)	129 (37.1%)	4 (50.0%)		131 (38.6%)	2 (11.8%)	
N3 (>9)	44 (12.6%)	0 (0%)		38 (11.2%)	6 (35.3%)	
Stage IV	60 (17.2%)	3 (37.5%)		63 (18.6%)	0 (0%)	
Unknown	15 (4.3%)	0 (0%)		15 (4.4%)	0 (0%)	
**Molecular subtype**						
TNBC	61 (17.5%)	5 (62.5%)	0.006	63 (18.6%)	3 (17.6%)	0.64
HR+	188 (54.0%)	3 (37.5%)		180 (53.1%)	11 (64.7%)	
HER2+	99 (28.4%)	0 (0%)		96 (28.3%)	3 (17.6%)	
**DFS**						
Median (Range)	31.0 [0, 335]	6.50 [0, 91.0]		29.0 [0, 335]	45.0 [10.0, 125]	
≤12 months	104 (29.9%)	5 (62.5%)	0.36	106 (31.3%)	3 (17.6%)	0.04
12-24 months	51 (14.7%)	1 (12.5%)		52 (15.3%)	0 (0%)	
24-36 months	40 (11.5%)	1 (12.5%)		37 (10.9%)	4 (23.5%)	
36-60 months	60 (17.2%)	0 (0%)		54 (15.9%)	6 (35.3%)	
>60 months	93 (26.7%)	1 (12.5%)		90 (26.5%)	4 (23.5%)	

BRCA1 tumors were more frequently grade 3 (P=0.03) as shown in (**Table 2** and **Figure 4C**), and FANCD2 mutations were also found more frequently in high-grade tumors (**Figure 4C**). Interestingly, when reviewing patients with family history of cancers, we found that these patients were more likely to harbor hereditary deleterious mutations in genes involving in homologous recombination DNA repair pathways including BRCA1, BRCA2, CHEK2, BARD1, FANCC, FANCM, and RAD50 than patients without family history of cancer (**Figure 4D** and **Table 2**).

When further examining associations between various clinical variables and deleterious gene mutations within each cancer subtype, we found that BRCA2 mutations were more prevalent in patients in HR+, Her2- and TNBC subtype patients. Patients with family history of cancers were more likely to be BRCA1 carriers (**Table 2**).

### Association of BRCA1/2 and DDR Deleterious Mutations With Clinical Outcome

**Figure 5:**
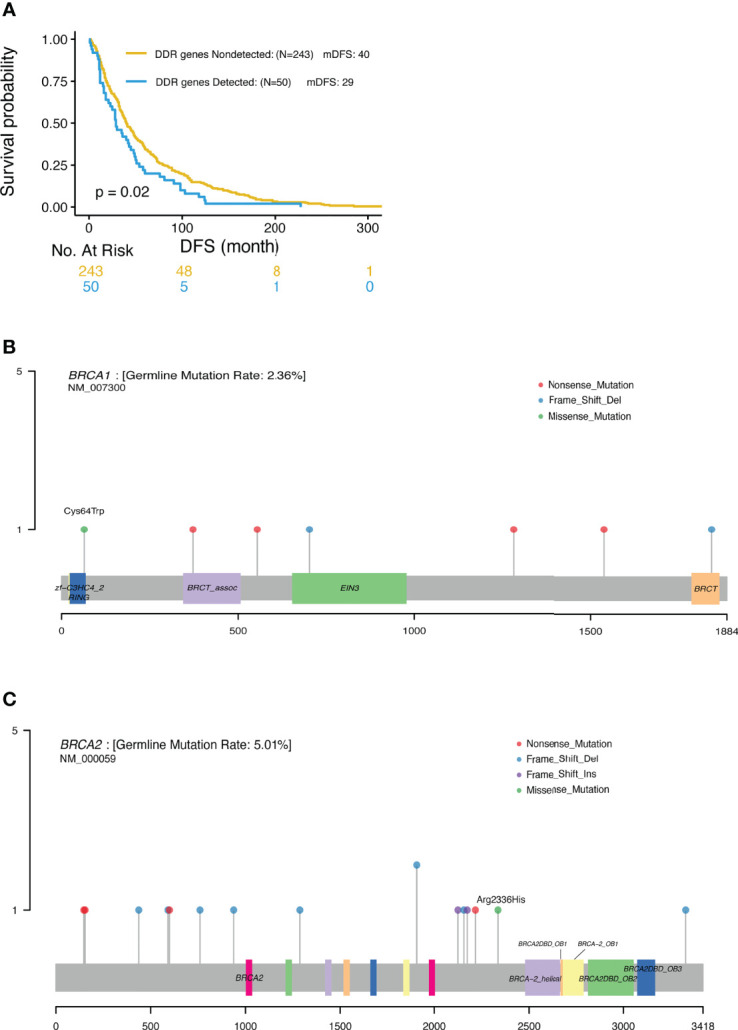
**(A)** Significantly different outcome associated with DDR gene mutations for DFS. **(B)** Locations of deleterious BRCA1 mutations. **(C)** Locations of deleterious BRCA2 mutations.

BRCA 1/2 mutations were observed in 7.6% and 4.5% TNBC patients, respectively (**Table 3**). In this study, only 2 out of the 25 patients with BRCA1/2 mutation received platinum-containing adjuvant treatment. The DFS of one patient was 91m and another was 81m; and the mDFS of the rest 23 BRCA1/2 carriers was 35m (95%CI: 22-48, range 1-125m). Analysis revealed a trend of favorable overall survival for patients with BRCA1/2 deleterious mutations, especially for triple negative breast cancer patients, although this trend was not statistically significant. Interestingly, when taking account of other genes in DNA damage repair, mutations in DDR genes including BRCA, ATM, RAD50, FANCD2 and CHEK2 were associated with shorter DFS (mDFS 29 vs 40 m, p=0.02, **Figure 5A**).

**Table 3:** Mutation rates of BRCA1/2 in different molecular subtypes and family history.

	No. of patients	BRCA1			BRCA2		
		N	%	P value	N	%	P value
**Molecular subtype**							
TNBC	66	5	7.6	0.006	3	4.5	0.64
HR+	191	3	1.6		11	5.8	
HER2+	99	0	0		3	3.0	
**Family History**							
Breast/ovarian cancer	35	1	2.9	0.04	4	11.4	0.17
Other cancers	64	4	6.3		2	3.1	
No	257	3	1.2		11	4.3	

## Discussion

In this study, NGS was used to profile genomic DNA collected from real world Chinese patients diagnosed with advanced breast cancer in order to identify the presence of cancer-associated germline mutations. Our analysis detected 87 pathogenic or likely pathogenic germline mutations across 77 patients. The most frequently mutated genes were BRCA2 (4.8%) and BRCA1(2.2%), consistent with previous data reported for Asian populations. Xie et al. reported that the prevalence rate of BRCA1/2 gene mutations in breast cancer patients in the Chinese population was 5.3% overall (3.5% in BRCA2 and 1.8% in BRCA1) (4). Similar frequencies were also previously reported in unselected Japanese breast cancer patients, with BRCA2 mutations observed in 2.71%, and BRCA1 mutations observed in 1.45% of the patients (17). These rates are lower than that observed in Ashkenazi Jewish patients (11.1%) (5) and Arab patients (10.2%) (19). Most of these deleterious mutations were nonsense (30) or frameshift (34) mutations, leading to premature proteins. In keeping with previous reports by others, pathogenic mutations were more commonly nonsense and frameshift mutations but less frequently missense mutations (5, 8, 10). As previously reported, around 80% of (likely) pathogenic mutations generated a premature termination codon truncating the encoded protein and 10% were missense variants encoding a stable mutant protein (20). Missense mutations classified as pathogenic tend to occur in limited regions of the BRCA proteins, including the RING domain and tandem BRCT domain in BRCA1, or the OB folds and helical domain in BRCA2 (20). In this study, we also detected a pathogenic missense mutation in BRCA1 (p. Cys64Trp) located in the RING domain (**Figure 5B**), and a pathogenic missense mutation in BRCA2 (p.Arg2336His) located in the upstream region of the helical domain (**Figure 5C**). Thus, in keeping with previous reports, the pathogenic BRCA1/2 mutations identified in this study were mostly located within the functional domains of these genes.

In this patient cohort, BRCA1 mutations were significantly associated with the triple negative phenotype relative to other BC subtypes. BRCA2 mutations were also more frequent in the triple negative breast cancer and HR+ HER2- subtypes, although these trends were not significant. These findings are consistent with data reported by others showing that triple negative breast cancers had the highest prevalence of deleterious gene mutations among the four molecular breast cancer subgroups (4), and that mutation rates of BRCA1 and BRCA2 were higher in TNBC compared to other breast cancer subtypes (10). Due to lack of known targets, TNBC is treated primarily with chemotherapy and is associated with poor survival (5). TNBC is a heterogeneous subtype (21), which Shao et al. has further classified into four transcriptome-based subtypes: (1) luminal androgen receptor (LAR), (2) immunomodulatory, (3) basal-like immune-suppressed (BLIS), and (4) mesenchymal like (22). These four TNBC subtypes exhibit different responses to treatment and survival (22). BLIS patients have been characterized with higher HRD scores compared with patients of other subtypes and therefore might benefit from DNA-damaging agents (22). Xie et al. reported that the frequency of BRCA1/2 mutations in TNBC was 11.2%, with BRCA1 in 7.4% and BRCA2 in 3.8% of patients respectively (4). In another prior study, BRCA1-positive mutation status was also strongly associated with TNBC (8). Moreover, 66-70% of BRCA1 carriers develop TNBC compared to 19-23% of BRCA2 carriers and 24% of non-carriers (7, 21), while 76% of BRCA2 mutation carriers develop HR+ HER2- disease (7). PALB2, and FANCM gene mutations have also previously been reported in association with the TNBC phenotype (21). Thus, the observed enrichment of BRCA1/2 germline mutations in TNBC, and BRCA2 germline mutations in HR+ patients in the present study of Chinese patients is consistent with previously reported findings across other patient groups. No other significant associations were observed between BC subtypes and any other genes in this study.

BRCA1/2 mutation status was also associated with age in our study. The average age of the patient cohort was 48.8 years, and a high proportion of deleterious BRCA2 mutations (23.5%) was detected in patients under 30 years. This finding is in keeping with other studies reporting that breast cancer patients with early onset age (under 40 years) were more likely to harbor deleterious mutations in BRCA1/2 (6.4%) and other DNA repair genes (4.5%) than those diagnosed at the age of 40 or later (4, 9). Moreover, the onset age in BC patients with DNA repair gene mutations has been reported to be significantly younger than that of non‐carriers (9). Breast cancer patients carrying BRCA1 or BRCA2 germline mutations have a mean onset age of 40 and 43 years, respectively, and patients carrying PALB2 mutations have a mean onset age of 53 years (21). Thus, similar to other populations, BRCA1/2 mutations were more prevalent in younger patients in our Chinese cohort.

Our study also found that BRCA1 tumors are more frequently high-grade, in keeping with previous publications reporting BRCA1 mutation carriers had tumors characterized for high histological grade and higher proportion of Ki67-positive cells than noncarriers (7, 8). In contrast to a previous report showing that axillary node involvement was more frequent in BRCA2 carriers (7), in our study BRCA2 mutations were more frequently found in patients without lymph node metastasis (**Table 2**).

In the present study, patients with family history of cancers were also more likely to harbor BRCA1/2 mutations (7.7%/13.3%) than patients without family history (1.6%/5.3%). Xie et al. reported that the rate of BRCA1/2 mutations in patients with familial breast cancer was 18.1% (4, 9). Other studies have revealed that relative to other types of cancer, BC is more commonly associated with a positive family history, followed by ovarian, colorectal, gastric, and cervical cancer (10). In one study, the proportion of cancers associated with positive family histories was 22.0% for all cancers, 7.2% for breast cancer, and 6.5% for breast and/or ovarian cancer (8). Moreover, a Japanese study found that for breast cancer patients, the frequency of family history of different cancer types associated with hereditary breast cancer syndromes was: 11.8% breast, 1.2% ovary, 3.5% pancreas, 2.9% prostate, and 0.8% thyroid cancer (17). Thus, our observation that BRCA1/2 germline mutations are enriched in Chinese breast cancer patients with a positive family history of cancer is consistent with patterns observed in other studies.

For breast cancer patients, there are conflicting results regarding the prognostic and predictive value of BRCA1/2 germline mutations (7). In a large unselected Chinese population, BRCA1 mutation carriers had significantly worse disease-free survival and disease-specific survival than noncarriers, while no significant difference in survival was found between BRCA2 mutation carriers and non-carriers (4). Other studies showed that Her2- BC patients with BRCA1/2 mutation had improved survival due to better response to treatment (5, 6, 23). In this cohort, two patients harboring BRCA1/2 mutation treated with platinum-containing adjuvant treatment, and obtained longer DFS. Germline mutations in BRCA1/2 and other DNA repair genes are associated with an increased risk of breast, ovarian, prostate and/or pancreatic cancer (24). Germline heterozygous mutations affecting BRCA2 also significantly elevate the risk of cancers of the pancreas, male breast, prostate, and other tissues (20). BRCA1/2 mutation carriers with different cancers shows different survival outcomes. Among ovarian cancer patients, BRCA1 and especially BRCA2 carriers respond better than non-carriers to platinum-based chemotherapy and have prolonged survival (7, 21, 25). BRCA1/2 mutation carriers also benefit from PARP inhibitors, and our previous study demonstrated that fluzoparib, a PARP inhibitor, had antitumor activity in BC and ovarian cancer, particularly in BRCA1/2-mutated patients (26).

In the present study, we observed a trend of favorable overall survival for BRCA1/2 carriers, most notably in TNBC patients, although this trend was not statistically significant. However, DDR pathway deficiency, encompassing alterations in a wider index of DNA damage response genes was significantly associated with shorter DFS and OS in carriers. Common deleterious mutations in other DDR genes besides BRCA1/2 including ATM, RAD50 and BARD1were also detected. However, these deleterious mutations were only detected in less than 2% of these patients. Mutations of non-BRCA1/2 genes in the HRR, DNA damage response and mismatch repair pathways have been reported to have medium-to-high penetrance of hereditary BC (8, 10). The rate of non-BRCA1/2 gene repair mutations is 6.5% vs 8.5% (8, 10). Most of these mutations are found in the ATM, CHEK2, PALB2, TP53, RAD50, RAD51D and BRIP1 genes (9, 10). Non-BRCA1/2 cancer-associated gene (RAD51D, TP53, MSH6, CHEK2, APC, and FANCC) mutation rate is 2.7% in Ashkenazi Jewish (16). PALB2 (1.2%) was the most commonly mutated gene other than BRCA1/2 in Chinese breast cancer patients, while CHEK2 (2.82%), ATM (1.06%), and PALB2 (0.87%) were the most commonly mutated genes other than BRCA1/2 in European populations (8). In this study, the most commonly mutated genes other than BRCA1/2 were ATM (6%), RAD50 (6%) and BARD1 (3%), which was not completely consistent with the data previously reported. This may be explained by the low rate of the mutations in these genes and the relatively small number of patients in this study. We also found DDR deficiency patients had shorter DFS (**Figure 5**), and these patients may benefit from treatment with PARP inhibitors. Overall, it appears that BRCA1 carriers have poorer survival, probably due to the fact that they frequently develop TNBC, whereas BRCA2 germline mutations were not found to have a prognostic impact. This poses a warrant of genetic testing for TNBC patients where PARP inhibitors can be added to improve treatment efficacy and prolong the survival of patients with DDR mutations. Based on our knowledge and understanding from testing results of this study, we designed some clinical trials using PARP inhibitors. There is an ongoing phase I trial of “Niraparib Plus Anlotinib in Advanced Solid Tumors with Homologous Recombination Repair (HRR) Gene Mutations (NCT04764084)” (27); and a “phase I trial to evaluate the safety and efficacy of fluzoparib in combination with apatinib in patients with OC or BC (NCT03075462)” has been finished and the result will publish soon.

## Limitations

i)We do not treat patients depend on the phenotype of the tumor varies whether it is a BRCA1 (mainly TNBC) or a BRCA2 (mainly HR positive) mutations; ii) Adjuvant therapy is a standard systemic treatment and depends on tumor stage, grade, and molecular subtypes, but not gene mutation variants.

## Conclusions

This was a comprehensive cancer-related genetic profiling analysis of a large cohort of Chinese patients with advanced breast cancer. Distinct distributions of pathogenic mutations in breast cancer subtypes and differential associations between mutation status and clinical features were observed. The most prevalent germline mutations were BRCA1/2 mutations, followed by ATM and RAD50 mutations. Collectively, our findings confirm the presence of BRCA1/2 and other DDR gene alterations in a substantial proportion of the Chinese breast cancer population and demonstrate their association with poor patient outcomes. Our observations verify the enrichment of these alterations among cancer patients diagnosed with TNBC and high-grade tumors, as well as in patients who are diagnosed at young age and/or have a positive family history of cancer. Our findings support the use of mutational profiling of Chinese breast cancer patients with these characteristics to assess the presence of germline mutations in BRCA1/2 and other DDR pathway genes in order to identify patients who may benefit from treatment with PARP inhibitors.

## Data Availability Statement

The data analyzed in this study was obtained from raw sequencing database call files (.BCL), the following licenses/restrictions apply the NGS data cannot be uploaded to a repository policy from Human Genetic Resource Administration of China(HGRAC). Requests to access these datasets should be directed to Xiaomei Wu (wuxiaomeihx@163.com).

## Ethics Statement

The studies involving human participants were reviewed and approved by IRB of Peking University Cancer Hospital. The patients/participants provided their written informed consent to participate in this study.

## Author Contributions

JZ, NW, and TZ contributed equally to this work. All authors contributed to the article and approved the submitted version.

## Conflict of Interest

Authors TZ, TL, YH, FX, YZ, SJ and JY were employed by Huidu Shanghai Medical Sciences.

The remaining authors declare that the research was conducted in the absence of any commercial or financial relationships that could be construed as a potential conflict of interest.

## Publisher’s Note

All claims expressed in this article are solely those of the authors and do not necessarily represent those of their affiliated organizations, or those of the publisher, the editors and the reviewers. Any product that may be evaluated in this article, or claim that may be made by its manufacturer, is not guaranteed or endorsed by the publisher.
