# Supplementary material for: Germline Mutational Landscape in Chinese Patients With Advanced Breast Cancer
Source: Front Oncol. 2022 Apr 13;12:745796. doi: 10.3389/fonc.2022.745796 (PMC9043949; doi:10.3389/fonc.2022.745796)
Supplement: Supplementary file 2 [file Table_1.docx]

| **Supplementary table. 64 DDR gene mutations detected in 57 patients** | | | | |
| --- | --- | --- | --- | --- |
| DDR gene | No. of TNBC | No. of HR+ | No. of Her2+ | Total |
| ABRAXAS1 | 0 | 1 | 0 | 1 |
| ATM | 0 | 3 | 2 | 5 |
| ATR | 1 | 0 | 0 | 1 |
| BARD1 | 2 | 1 | 0 | 3 |
| BRCA1 | 5 | 3 | 0 | 8 |
| BRCA2 | 3 | 11 | 3 | 17 |
| BRIP1 | 0 | 1 | 0 | 1 |
| CHEK2 | 0 | 2 | 0 | 2 |
| ERCC2 | 0 | 0 | 1 | 1 |
| FANCC | 2 | 0 | 0 | 2 |
| FANCD2 | 1 | 2 | 0 | 3 |
| FANCE | 0 | 0 | 1 | 1 |
| FANCG | 0 | 1 | 0 | 1 |
| FANCL | 1 | 0 | 0 | 1 |
| FANCM | 0 | 1 | 1 | 2 |
| MRE11 | 1 | 0 | 0 | 1 |
| NBN | 0 | 1 | 0 | 1 |
| PMS2 | 0 | 2 | 0 | 2 |
| RAD50 | 1 | 3 | 1 | 5 |
| RAD51C | 0 | 1 | 0 | 1 |
| RAD51D | 0 | 0 | 1 | 1 |
| STK11 | 1 | 0 | 0 | 1 |
| TP53 | 0 | 1 | 1 | 2 |
| XRCC1 | 0 | 1 | 0 | 1 |

*7 patients were detected 2 DDR gene mutations.

**7 patients were de novo stage IV.
